# LTF Regulates the Immune Microenvironment of Prostate Cancer Through JAK/STAT3 Pathway

**DOI:** 10.3389/fonc.2021.692117

**Published:** 2021-11-10

**Authors:** Qi Zhao, Yingying Cheng, Ying Xiong

**Affiliations:** Department of Urology, The First Affiliated Hospital of Yangtze University, Jingzhou, China

**Keywords:** prostate cancer (PRAD), tumor-infiltrated immune cells (TIICs), lactoferrin (LTF), granulocyte-macrophage colony-stimulating factor (GM-CSF), JAK/STAT3

## Abstract

**Background:**

The study of the immune microenvironment in prostate cancer (PRAD) has brought new opportunities for the current traditional treatment regimens. Therefore, our goal is to develop a universal immunodiagnostic marker to improve patient survival.

**Methods:**

Bioinformatics analysis: We collected 591 samples from The Cancer Genome Atlas (TCGA) and Gene Expression Omnibus (GEO) cohorts and evaluated the abundance and distribution of immune cell members in the PRAD expression profile matrix in the mixed cell population by CIBERSORT, ESTIMATE, single-sample gene set enrichment analysis (ssGSEA), and other methods. The target genes related to PRAD immune microenvironment and tumor mutation load were obtained by overlap analysis and verified by pan-cancer analysis. Cell experiment: The cell transfection scheme was designed, and the experiment was divided into three groups: overexpressing lactoferrin (LTF) group, empty plasmid group, and control group. After obtaining cells in each group, the gene and protein expression levels of LTF and signal transduction of signal transducer and activator of transcription 3 (STAT3) and granulocyte-macrophage colony-stimulating factor (GM-CSF) in the above three groups were detected by real-time PCR and Western blot, respectively. Finally, the level of GM-CSF secretion in the three groups was detected by ELISA.

**Results:**

Macrophages, resting mast cells, and plasma cells play an important role in PRAD immune microenvironment. In addition, high tumor mutation load [tumor mutational burden (TMB)] was positively correlated with lymph node metastasis in patients with PRAD. As the core gene of the PRAD immune microenvironment, the low expression of LTF in PRAD promotes the occurrence of immunodeficiency, PRAD, and the enrichment of the Janus kinase (JAK)/STAT3 signal pathway. Through cell experiments, it was found that the content of LTF mRNA and protein increased significantly, while the content of STAT3 and GM-CSF mRNA and protein decreased significantly in the overexpressed LTF group. The level of GM-CSF in the supernatant of cell culture was significantly decreased in the overexpression group of LTF.

**Conclusion:**

The core gene we proposed is one of the most promising biomarkers to improve the overall survival rate of PRAD and provides an important theoretical basis for the study of the mechanism of the LTF-mediated JAK/STAT3 pathway in PRAD.

## Background

Prostate cancer (PRAD) is a common male cancer in the world. Advanced PRAD has a poor prognosis and usually progresses to androgen-resistant prostate cancer [castration-resistant prostate cancer (CRPC)] (1). However, at present, there is no radical treatment for CRPC, and the latest data show that the median overall survival time of CRPC is only 12.2–34.7 months (2).

Tumor immune system-targeting therapy is considered to be an important direction in the development of anticancer drugs (3). In recent years, new immune system therapies, such as immune checkpoint blocking, cancer vaccines, and targeted antibodies, have shown significant effects in the treatment of tumors (4). SiPuleucel-t, a personalized tumor vaccine against prostatic acid phosphatase (PAP), has become the first immune agent approved by the Food and Drug Administration (FDA) to treat CRPC. Therefore, a large number of vaccine experiments against prostate-associated antigens have become a research hotspot (5). In recent years, with the great advantages of bioinformatics in T-cell receptor sequencing, immune microenvironment assessment, and new antigen prediction, it has brought new ideas for current immunotherapy (6).

At present, there is no systematic evaluation of the immune microenvironment of PRAD based on bioinformatics methods and the construction of related biological pathway models. If only a small number of samples are used to study one or two types of tumor-infiltrating immune cells (TIICs) or only a single bioinformatics method is used without sufficient experimental verification, unreliable results may be produced. Therefore, our project is based on a large number of datasets using bioinformatics methods and cell experiments to confirm the changes of the PRAD immune microenvironment, identify the core genes that affect the microenvironment, and explore the mechanism of the gene-mediated pathway. The flowchart of this study is shown in **Supplementary Figures S1**, **S2A–D**).

## Materials and Methods

### Data Collection

The datasets supporting the conclusions of this article are available in the Gene Expression Omnibus (GEO) and The Cancer Genome Atlas (TCGA) repository (https://portal.gdc.cancer.gov/projects/TCGA/PRAD, https://www.ncbi.nlm.nih.gov/geo/query/acc.cgi?acc=GSE3325, and https://www.ncbi.nlm.nih.gov/geo/query/acc.cgi?acc=GSE55945).

Clinical features, RNA sequencing (RNA-seq) expression data (tumor = 499, normal = 52), and somatic mutation information (n = 440) of patients with prostate tumors were collected from TCGA database. Clinical features include sex, age, TNM stage, survival status, and survival time. The other two independent PRAD verification sets GSE3325 and GSE55945 were obtained from GEO. Based on the GPL570 platform, a total of 40 samples, including 26 tumor samples and 14 normal samples, share common clinicopathological features.

The main materials for the cell experiments include PC-3 cells (PRAD cells, American tissue culture bank), fetal bovine serum (FBS; Hyclone, Logan, UT, USA); F12K medium, opti-MEM medium (GIBCO, Grand Island, NY, USA); trypsin (Gibco, Grand Island, NY, USA); penicillin (Sigma-Aldrich, St. Louis, MO, USA); polyvinylidene fluoride (PVDF) membrane (0.45 μm) (Millipore, Schwalbach, Germany); pre-stained protein marker (Green BioResearch, LA, USA); Lichunhong, Tween 20, acrylamide, sodium dodecyl sulfate (Solon, OH, USA); protein lysate [radioimmunoprecipitation assay (RIPA); Biyuntian Institute of Biotechnology, Beyotime Biotechnology, Shanghai, China]; anti-lactoferrin (LTF) antibody (ab109216), anti-signal transducer and activator of transcription 3 (STAT3) antibody (ab119352), anti-granulocyte-macrophage colony-stimulating factor (GM-CSF) antibody (ab193345); Lipofectamine 2000 transfection reagent (Thermo Fisher Scientific, NY, USA); Prime STAR HS DNA polymerase (Takara, Beijing, China); reverse transcription kit (1708843; Bio-Rad Laboratories, CA, USA); primer synthesized by Nanjing Jinsi Rui Biotechnology Co., Ltd.; pIRESneo3 vector (youBio, Changsha, China); AFL II, Not I [New England Biolabs (Beijing) Ltd., Beijing, China]; and human GM-CSF Quantikine ELISA Kit (DGM00; R&D Systems, MN, USA).

### Analysis of Immune Cell Infiltration in the Microenvironment of Prostate Cancer

The R software CIBERSORT package (version 3.6.3) was used to evaluate the relative expression levels of 22 infiltrating immune cell types (TIICs) in PRAD samples, namely (7, 8), T cells (CD8+ T cells, CD4+ T cells, resting memory CD4+ T cells, naive CD4+ T cells, γδ T cells, regulatory T cells, and follicular helper T cells), B cells (naive and memory B cells and plasma cells), natural killer (NK) cells (activated and resting NK cells), and myeloid subsets (M0 macrophages, M1 macrophages, M2 macrophages, activated and resting mast cells, activated and resting dendritic cells, neutrophils, monocytes, and eosinophils). We evaluated the fractions of these 22 TIIC subpopulations in each sample. Then, we validated using the GSE3325 and GSE55945 datasets.

### Analysis of Immune Microenvironment Based on Single-Sample Gene Set Enrichment Analysis

Single-sample gene set enrichment analysis (ssGSEA) (http://software.broadinstitute.org/gsea/msigdb/index.jsp) has the function of classifying gene sets of common biological function, chromosome mapping, and physiological regulation (9, 10). We used ssGSEA to quantify the activity or enrichment level of immune cells, functions, or pathways in cancer samples and to predict standardized enrichment scores for each immune category in a single tissue sample. Patients with PRAD were divided into three groups according to the clustering results of enrichment scores. The three groups of PRAD patients were scored by the ESTIMATE algorithm (11, 12) and divided into high, medium, and low immune evaluation groups.

### Analysis of Tumor Mutation Load in Somatic Mutation Samples

It is reported that highly mutated tumors are more likely to carry new antigens, making them targets for activating immune cells (13). A large number of new antigens are related to the response to immunotherapy, while tumor mutational burden (TMB) is similar to the number of new antigens potentially recognized by the immune system (14, 15). Therefore, we explore the possibility of immunotherapy for PRAD based on TMB. We visualized the somatic mutation data of PRAD, calculated the TMB value of each sample, and analyzed the relationship between TMB value and lymph node metastasis. According to the median TMB value of each sample, patients with PRAD were divided into high TMB groups and low TMB groups. Wilcox test method was used to analyze the differences to screen the genes that meet the criteria.

### Acquisition of Target Gene

First of all, immune-related genes were retrieved from the IMMPORT database (https://www.immport.org/shared/genelists) (16), and the candidate genes were obtained by VENN analysis with the above-obtained differential genes. Finally, based on the TNM staging data of PRAD, the candidate genes were analyzed by univariate logistic regression analysis, and the key genes were screened. Therefore, it can be considered that this gene is the most likely immune-related target gene in the progression of PRAD.

### Target Gene Verification Based on Pan-Cancer Analysis

We downloaded 33 main tumor transcriptome data [fragments per kilobase of transcript per million map reads (FPKM)], mutation data (varscan), and clinical data from TCGA database from the UCSC Xena website and used Perl software to extract the LTF expression matrix for the following analysis. The specific tumors are shown in **Supplementary Table S1**.

Wilcoxon test was used to analyze the difference of LTF expression matrix between normal group and tumor group (Tumor = 10,327, Normal = 730), and the results were visualized. The LTF expression level of each patient was combined with progression-free interval (PFI) data, and the samples were divided into high and low groups according to the median level of LTF expression. The PFI survival curves of the two groups were analyzed by the Kaplan–Meier method.

The LTF values and microsatellite instability (MSI) values of 33 kinds of tumor somatic mutation samples (n = 10,114) were calculated. The correlation between LTF expression, TMB value, and MSI value was tested by the Spearman method, and then the correlation radar map of TMB and MSI was drawn. The microenvironment scores of all samples were calculated and combined with LTF expression to test the correlation between LTF expression and immune microenvironment score.

### The Enrichment Analysis of the LTF Gene

To explore the potential molecular mechanisms of the screened target genes, we performed gene enrichment analysis, including Kyoto Encyclopedia of Genes and Genomes (KEGG) analysis and Gene Ontology (GO) analysis. The repeat number of GSEA software was set to 1,000; P-value <5%.

### Cell Culture

PC-3 cells were subcultured in Dulbecco’s modified Eagle’s medium (DMEM) containing penicillin (final concentration 100 U/ml), streptomycin (final concentration 100 μg/ml), and 10% FBS. All the cells were incubated in a humidified atmosphere of 5% CO_2_ at 37°C.

### Plasmid Construction and Transfection

The RNA of PC-3 cells was extracted and reverse transcribed to obtain cDNA and used in the PCR amplification experiment. All the primers used followed the principles of primer design. The specific primers can be found in **Supplementary Table S2**. Colony PCR was used to identify positive clones. Twenty-four hours before transfection, the cells in the logarithmic growth phase were digested with trypsin, and the cell density was adjusted in the medium containing 10% serum (without antibiotics). The cells were re-inoculated in a 24-well plate at 37°C and 5% CO_2_. Lipofectamine 2000 was used for transfection. According to Lipofectamine 2000 instructions, the experiment was divided into three groups: Control group: normal culture of PC-3 cells; pIRESneo3-Control group: transfection of PC-3 cells with pIRESneo3-Control plasmid; pIRESneo3-LTF group: transfection of PC-3 cells with pIRESneo3-LTF plasmid. After transfection, the cells were cultured at 37°C and 5% CO_2_ incubator for 6 h and then replaced with the medium containing 10% FBS. Forty-eight hours after transfection, the follow-up experiment was carried out.

### RNA Isolation and Reverse Transcription-Quantitative Polymerase Chain Reaction

Total RNA was separated by TRIzol reagent, and RNA samples were reverse transcribed into cDNA by RNA reverse transcription kit. The qPCR was performed on the LightCycler 480 Real-Time PCR System. Glyceraldehyde 3-phosphate dehydrogenase (GAPDH) mRNA was used as an internal control for each sample, and the expression of each sample was normalized to GAPDH mRNA. The reaction parameters were set and amplified, and the results were calculated by 2-^ΔΔCT^, and the primers used were found in **Supplementary Table S3**.

### Western Blot Analysis

Total protein was extracted from the transfected cells 48 h after transfection, and protein concentration was determined by the bicinchoninic acid (BCA) protein assay kit. The whole protein samples were loaded onto 10% sodium dodecyl sulfate polyacrylamide gel electrophoresis (SDS-PAGE) gels and were transferred to a PVDF membrane after electrophoresis. The membrane was blocked with 5% fat-free milk and then incubated with anti-LTF antibody (1:5,000; ab109216), anti-STAT3 antibody (1:5,000; ab119352), and anti-GM-CSF antibody (1:500; ab193345) overnight at 4°C, followed by peroxidase-conjugated secondary antibody (1:5,000) incubation for 1 h at room temperature. The luminous imaging workstation Tanon 6600 was used for detection, and the optical density (OD) was analyzed by Image-Pro Plus 6.0 software. Finally, the statistical differences between the test groups were tested by one-way ANOVA and Tukey’s test; P < 0.05 was considered to have a significant difference.

### Enzyme-Linked Immunosorbent Assay

The supernatant of cell culture of each group was taken, and the expression level of GM-CSF in the supernatant was detected according to the instructions of the kit. The OD value was determined by an enzyme labeling instrument at 450 nm.

## Results

### The Immune Microenvironment of Prostate Cancer

**Figure 1:**
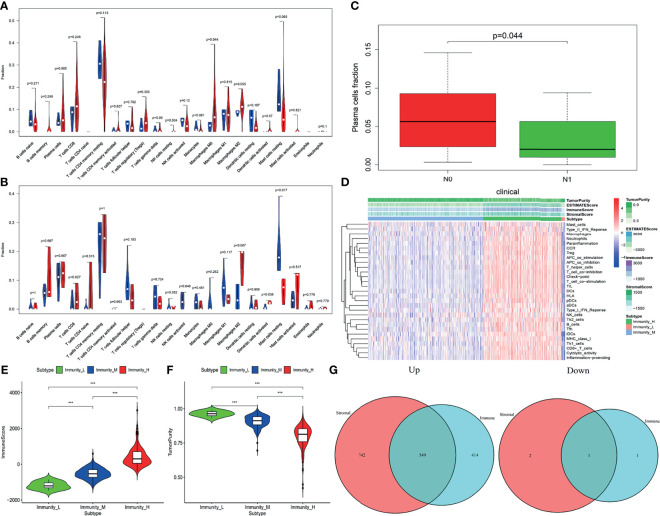
**(A, B)** The distribution and abundance difference of tumor-infiltrating immune cells (TIICs) in normal and cancerous tissues of the prostate. Red indicates tumor tissues, and blue indicates normal tissues [**A**, The Cancer Genome Atlas (TCGA) cohort; **B**, Gene Expression Omnibus (GEO) cohort]. **(C)** Correlation of infiltrating plasma cell fraction with clinical features in prostate cancer (PRAD) patients. N, lymph node, which represents the presence and extent of regional lymph node metastasis; N0 means that the lymph node is not involved, and N1 means that the lymph node is involved. **(D)** A heat map of the relationship between the proportion of 29 immune-related gene sets and tumor purity, microenvironment score (including matrix score and immune score), and sample immune subtype based on TCGA data. Red means upward regulation; blue means downward regulation. **(E, F)** Relationship between immune scores **(E)** and tumor purity **(F)** and sample immune subtypes. The relative numerical values corresponding to the height of the histogram indicate the different levels of abundance and the proportions (***P ≤ 0.001). **(G)** The upregulated and downregulated differential genes of stromal score groups and immune score groups were obtained, respectively (549 upregulated genes and one downregulated gene).

The proportion of macrophages M0 and M2 cell fractions in TCGA group was significantly higher than that in normal tissues. M2 macrophage cell fractions increased in the GEO group, while resting mast cell fractions decreased in TCGA and GEO groups (**Figures 1A, B**). Besides, the plasma cell ratio was positively correlated with lymphatic metastasis (**Figure 1C**).

Based on the cluster analysis of ssGSEA immune-related genomes, PRAD patients were divided into low, medium, and high immune activity groups according to the immune activity score. As shown in the figures, we found that the immune activity of patients was positively correlated with immune cell score and negatively correlated with tumor purity (**Figures 1D–F**).

A total of 549 upregulated genes and one downregulated gene were obtained by gene difference analysis of the PRAD immune microenvironment (**Figure 1G**).

### Tumor Mutational Burden of Prostate Cancer

**Figure 2:**
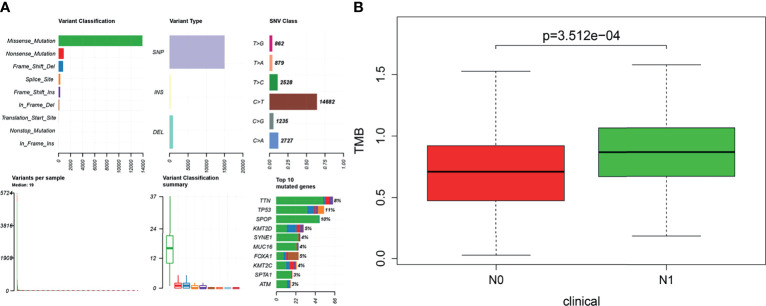
**(A)** Based on the R software “maftools” package (version 3.6.2), the somatic mutation data of prostate cancer were visualized to Variant Classification, Variant Type, SNV Class, and so on. **(B)** Tumor mutational burden (TMB) value was positively correlated with lymphatic metastasis in patients with prostate cancer (P < 0.001). The longitudinal coordinate is the relative value of TMB; N0 indicates no lymph node metastasis, and N1 indicates lymph node metastasis.

The visualization results of somatic mutation data of PRAD showed that the variant classification was mainly missense mutation, and the variant type was mainly single nucleotide polymorphism (SNP). The overall gene mutation rate of all samples was 59.92%, and the top 10 genes with mutation rates were TP53, SPOP, TTN, KMT2D, FOXA1, KMT2C, MUC16, SYNE1, ATM, and SPTA1 (**Figure 2A**). Also, we found that the TMB value of N1 was higher than that of N0 in the clinical features of TNM (**Figure 2B**). A total of 146 genes with statistical significance were obtained by analyzing the differences in expression profiles between the two groups.

### Key Genes Related to Prognosis in the Immune Microenvironment of Prostate Cancer

**Figure 3:**
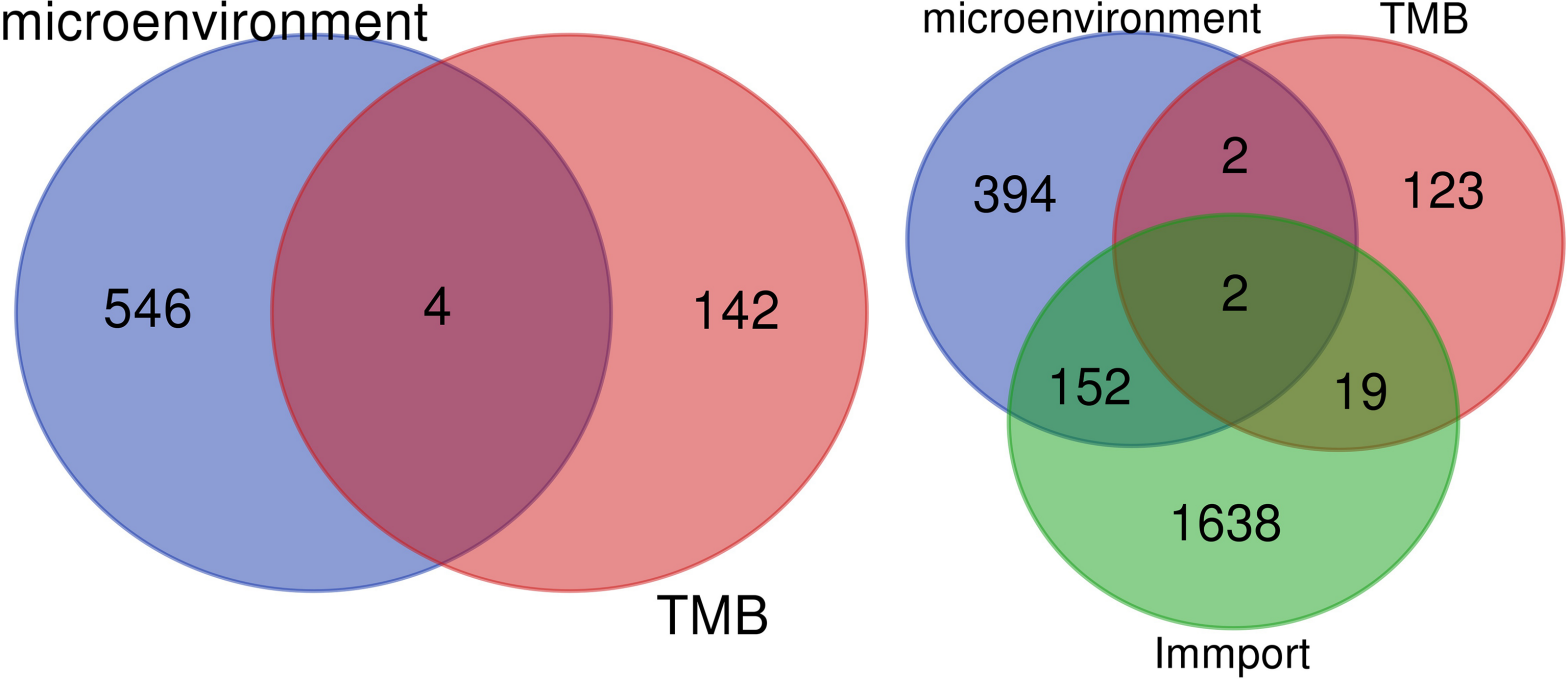
We retrieved 2,498 immune-related genes from the ImmPort database and obtained a Venn map of the immune microenvironment, tumor mutational burden (TMB), and immune-related genes based on the informatics and Evolutionary Genomics website.

The overlapping genes were obtained by VENN analysis of 550 differential genes obtained by tumor immune microenvironment grouping, 146 differential genes obtained by TMB value grouping, and 2,498 immune-related genes retrieved from the ImmPort database. Finally, two genes, CSF3 and LTF, which are most closely related to the immune microenvironment, were screened (**Figure 3**). Then, based on the clinical characteristics of the TNM stage of TCGA cohort, CSF3 and LTF were analyzed by multivariate logistic regression. The odds ratio (OR) values of LTF in N0/N1 were 0.641, P = 0.055, but CSF3 was not statistically significant. It is suggested that LTF is related to the progression and lymphatic metastasis of PRAD and can be used as a key gene affecting prognosis when evaluating the immune microenvironment of PRAD.

### Expression of Lactoferrin in 33 Tumor Types

**Figure 4:**
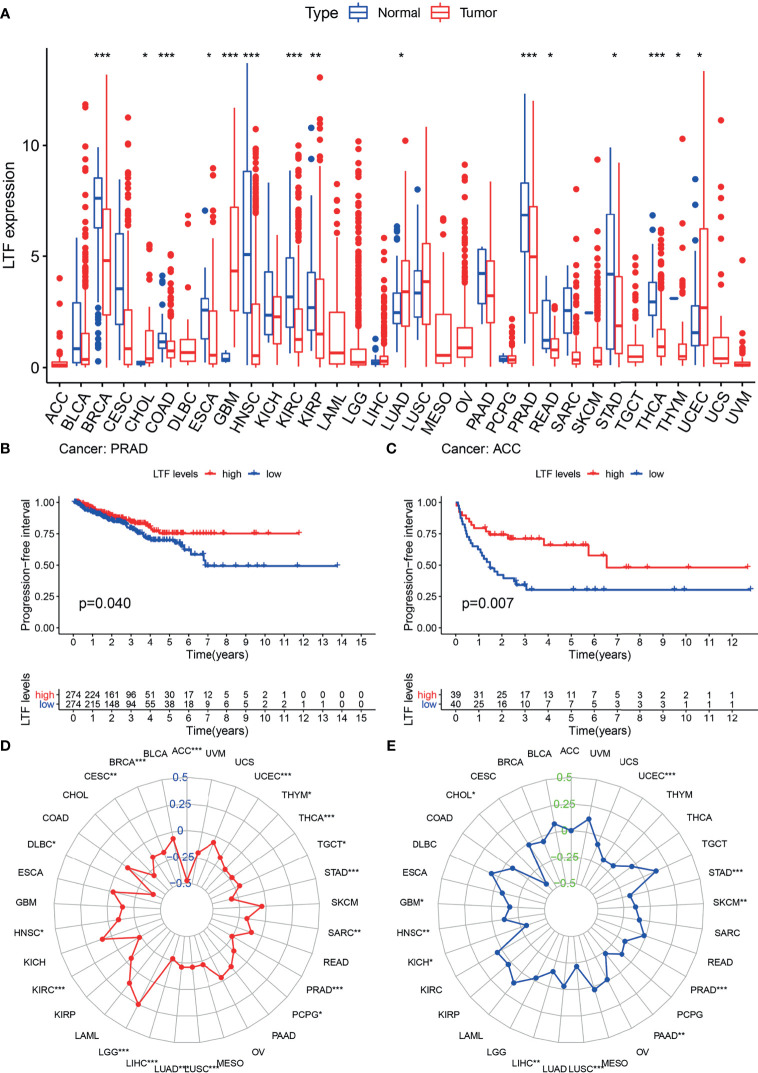
**(A)** The expression and distribution of lactoferrin (LTF) in cancer tissues and paracancerous normal tissues of 33 tumor types. Red indicates tumor tissues, and blue indicates normal tissues (***0.001, **0.01, *0.05, “ “1). **(B, C)** Kaplan–Meier method was used to analyze the progression-free interval rate of patients, and it was found that patients with high LTF expression group were significantly higher than those with low LTF expression group. **(D, E)** Tumor mutational burden (TMB) **(D)** and microsatellite instability (MSI) **(E)** correlation radar chart visualizes the TMB and MSI correlation of LTF in each tumor (***0.001, **0.01, *0.05, “ “ 1).

Differential analysis of 33 tumor types (**Supplementary Table S1**) showed that LTF was differentially expressed in 20 tumor types. Moreover, in most tumors, the expression level was higher in the normal group than in the tumor group (**Figure 4A**). This suggests that LTF expression is suppressed in most tumors.

The expression of LTF in ACC and PRAD was related to progression-free survival (**Figures 4B, C**). From the survival curve, it can be seen that the survival rate of patients with low expression of LTF is significantly lower than that of patients with high expression. It shows that the high expression of LTF makes the tumor remain in a state of continuous remission to a certain extent; that is, the symptoms are improved and the effect of chemotherapy is good.

The expression level of LTF in 33 tumor types was negatively correlated with the TMB value of 17 tumor types (including PRAD) and the MSI value of 11 tumor types (including PRAD). These results indicated that LTF has the function of maintaining genomic stability (**Figures 4D, E**).

### The Biological Function of Lactoferrin Enrichment

**Figure 5:**
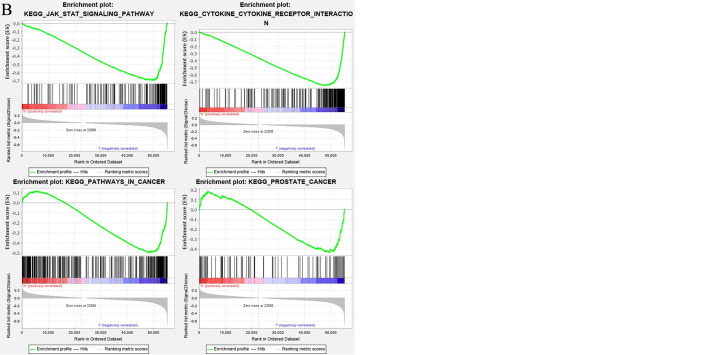
**(A)** The biological function enrichment analysis of lactoferrin (LTF) was performed by gene set enrichment analysis (GSEA) software, and Kyoto Encyclopedia of Genes and Genomes (KEGG) results showed that the high expression of LTF mainly promoted a variety of biological pathways, such as aminoacyl tRNA biosynthesis, mismatch repair, ubiquitin-mediated proteolysis, and nucleotide excision repair. **(B)** The biological function enrichment analysis of LTF was carried out by GSEA software. KEGG results showed that the high expression of LTF was related to the inhibition of biological pathways such as the Janus kinase (JAK)/signal transducer and activator of transcription 3 (STAT) signal pathway, tumor pathway, and prostate cancer progression pathway.

The enrichment of LTF was analyzed by GSEA software, and the KEGG results showed that the high expression of LTF was mainly concentrated in the promotion of biological pathways such as aminoacyl tRNA biosynthesis, mismatch repair, ubiquitin-mediated protein hydrolysis, and nucleotide excision repair (**Figure 5A**). The low expression of LTF is mainly concentrated in the promotion of biological pathways such as cytokine–cytokine receptor interaction, Janus kinase (JAK)/STAT signaling pathway, tumor pathway, and PRAD development pathway (**Figure 5B**).

### Effects of Lactoferrin Overexpression on mRNA and Protein Expression Levels of JAK/STAT3 Pathway and GM-CSF in PC-3 Cells

**Figure 6:**
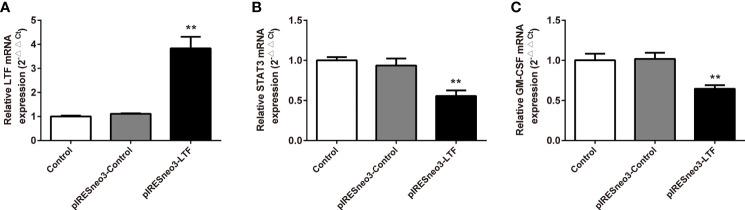
Effect of the mRNA expressions of LTF, STAT3 and GM-CSF in PC-3. The mRNA levels of LTF, STAT3 and GM-CSF were detected by RT-PCR. The mRNA levels of LTF **(A)**, STAT3 **(B)** and GM-CSF **(C)** were normalized to control. The results were presented as mean ± SD (n = 3). **p < 0.01 *vs*. pIRESneo3-Control group.

**Figure 7:**
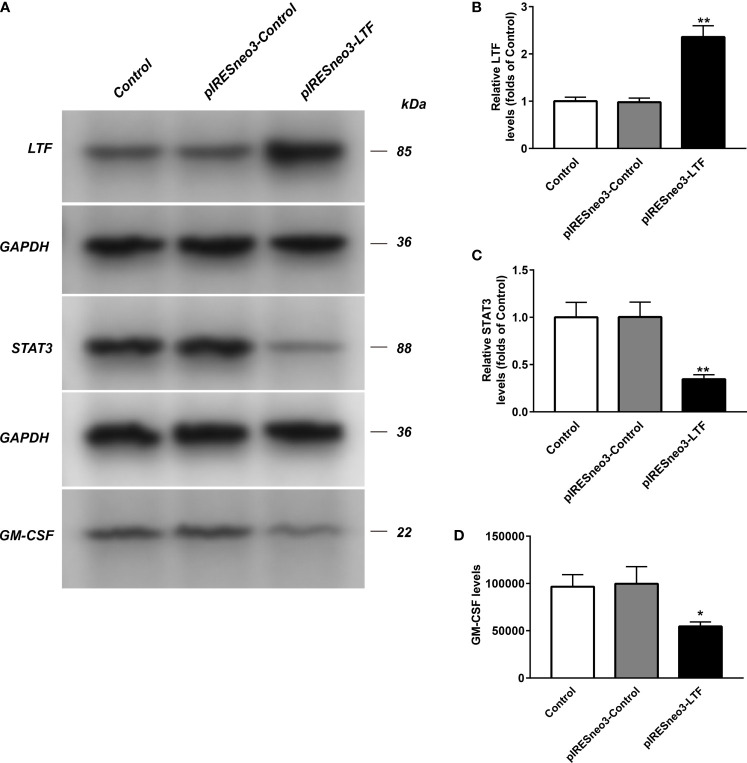
The LTF, STAT3 and GM-CSF expressions in PC-3 from different groups were detected by western blot assay, and representative bands were shown in **(A)**. The levels of LTF **(B)**, STAT3 **(C)** and GM-CSF **(D)** were normalized to control. The results were presented as mean ± SD (n = 3). *p < 0.015, **p < 0.01 vs. pIRESneo3-Control group.

The mRNA and protein levels of the transfected cells in the three groups were analyzed: the mRNA and protein expressions of LTF, STAT3, and GM-CSF were detected. As shown in **Figure 6**, compared with the pIRESneo3-Control group, the mRNA contents of LTF (P < 0.01) in the pIRESneo3-LTF group were significantly increased, while the mRNA contents of STAT3 (P < 0.01) and GM-CSF (P < 0.01) were significantly decreased. The differences were statistically significant. As shown in **Figure 7**, compared with the pIRESneo3-Control group, the protein content of LTF (P < 0.01) in the pIRESneo3-LTF group was significantly increased, while the protein content of STAT3 (P < 0.01) and GM-CSF (P < 0.015) was significantly decreased, with statistically significant differences.

### Effect of Lactoferrin on the Level of GM-CSF in the Culture Supernatant

**Figure 8:**
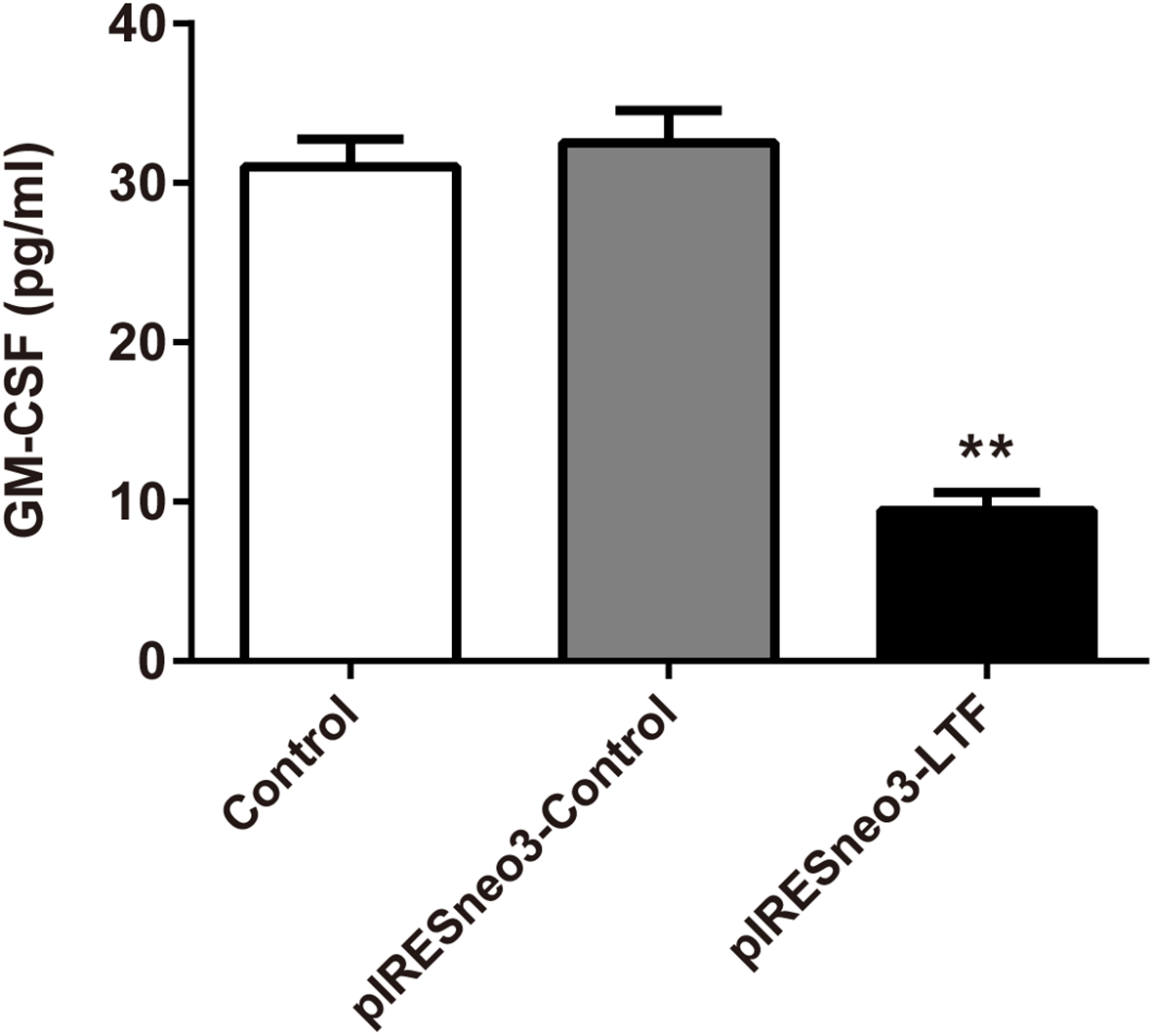
LTF overexpression downregulated GM-CSF level in PC-3. The culture supernatant was subjected to ELISA. The results were presented as mean ± SD (n = 3). **P < 0.01 *vs*. pIRESneo3-Control group.

The results of ELISA showed that the level of GM-CSF in the culture supernatant of the targeted overexpression LTF group was significantly lower than that of the negative control group (**Figure 8**).

### Lactoferrin Mediates JAK/STAT3 Pathway to Regulate the Secretion of GM-CSF

The above experiments found that LTF showed low expression in most tumors, including PRAD, while it showed high expression in normal tissues, indicating that LTF is a protective gene in PRAD. At the same time, our GSEA showed that the downregulation of LTF could inhibit the normal immune response *in vivo* and was closely related to the enrichment of the JAK/STAT3 signal pathway. We have confirmed that LTF plays an important leading role in the regulation of the immune microenvironment, and a large number of works of literature have confirmed that the production of GM-CSF is closely related to the tumor immune microenvironment. To further explore its mechanism, we overexpressed the LTF gene in PRAD PC-3 cells and verified the effect of high expression of LTF on JAK/STAT3 pathway-related proteins and PRAD-derived GM-CSF proteins by Western blot analysis, RT-qPCR, and enzyme-linked immunosorbent assay. The results show that overexpression of LTF may reduce the secretion of GM-CSF by tumor cells through JAK/STAT3 pathway to regulate the tumor immune microenvironment and inhibit tumor cell proliferation and migration.

## Discussion

Some studies have suggested that PRAD is an immunogenic disease and can be used as a model for studying therapeutic cancer immune-targeted therapy (17, 18), and immune cell infiltration in the tumor immune microenvironment is considered to play an important role in the biological behavior of many cancers (11, 19).

We found that a high immune cell infiltration score was significantly associated with poor prognosis in patients with PRAD, in which the proportion of infiltrating macrophages M0, M2 was significantly higher than that in normal tissues, while resting mast cells were lower than that in normal tissues. Also, plasma cell levels were positively correlated with tumor lymphatic metastasis. Some studies have shown that macrophages are an important part of the tumor microenvironment. M2 macrophages can alleviate inflammation and adaptive Th2 immunity and promote angiogenesis. In cancer, the tumor microenvironment makes the polarization balance of macrophages tend to M2 phenotype (20). In addition, it has been confirmed that dihydrotestosterone (DHT) increases the cytotoxic activity of macrophages through the upregulation of TNF-related apoptosis-inducing ligand (TRAIL), while castration induces the proliferation of androgen-resistant PRAD cells by reducing the activity of macrophages (21, 22). In the early stage of tumor development, mast cells inhibit tumor growth by chemotactic CD8+ T cells; with further development of the tumor, a large number of endogenous damage-associated molecular pattern (DAMP) molecules in the tumor microenvironment change the characteristics of mast cells; chemotactic regulatory T (Treg) cells infiltrate into the tumor area, thus promoting tumor growth (23). The study of plasma cells in human hepatocellular carcinoma (HCC) tissue by Wei et al. (24) showed that the increase of plasma cell level was related to the poor prognosis of patients. This is consistent with our results; that is, the proportion of M0, M2 macrophages in the PRAD immune microenvironment is significantly higher than that in normal tissues, androgen can activate macrophages, and the change of resting mast cell characteristics and the infiltration of a large number of plasma cells promote the proliferation of PRAD. Therefore, our study can guide the clinical practice of PRAD immune-targeted therapy and individualized therapy for patients with obvious immune cell infiltration.

High TMB is an effective indicator of new biomarkers sensitive to immune checkpoint inhibitors (25). We identified several genes with the highest mutation load in PRAD, such as TP53, SPOP, and KMT2. It has been reported that KMT2C and KMT2D are the third and seventh most common mutations in cancer genes, which may lead to more cancer cell initialization and metastasis (26). Therefore, further exploration of SPOP and KMT2 family mutant genes is helpful to understand the mechanism of PRAD cell proliferation.

The pan-cancer analysis confirmed the low expression of LTF in most tumors, including PRAD, and confirmed that LTF was a tumor suppressor gene. We found that there was a negative correlation between TMB, MSI, and LTF in urinary tumors (including PRAD) (P < 0.001). The lower the expression of LTF in tumors, the higher the level of MSI, TMB. MSI is an abnormal expression of mismatch repair gene system in DNA replication, which may be an important molecular event leading to the occurrence and development of tumor (27). Some studies have shown that MSI has a high incidence in low Gleason score or low serum prostate specific antigen (PSA), which plays a certain role in the progression of tumor and is a sign of a good prognosis of PRAD (28). Programmed cell death 1 (PD-1) antibody pembrolizumab is approved for the treatment of solid tumors with microsatellite high instability (MSI-H) or mismatch repair defect (dMMR) (29, 30). The use of proactive tumor sequencing to screen the expression levels of MSI-H/dMMR and LTF in all patients with advanced PRAD is of great significance for the personalized treatment of PRAD patients (31, 32). In addition, the PFI survival curve showed that the survival rate of patients with low expression of LTF was lower than that of patients with high expression of LTF, so it was speculated that maintaining high expression of LTF could lead to continuous remission of PRAD to some extent. The above results provide effective support for us to explore the role of LTF in the progression of PRAD. LTF can be used as an effective immune-related prognostic marker for PRAD.

We found that the high expression of LTF in tumors can promote mismatch repair, nucleotide excision repair, and other pathways, while the low expression of LTF is related to primary immunodeficiency, JAK/STAT signaling pathway, cancer pathway, PRAD, and other pathways’ enrichment. It can be concluded that LTF is a protective gene in PRAD, and its downregulation will inhibit the normal immune response in the body, thus making cancer cells escape the immune process. More importantly, it was found that there was a close relationship between the LTF gene and the JAK/STAT signal pathway in the immune microenvironment of PRAD. It was also reported that STAT3 phosphorylation was positively correlated with the decrease of survival rate. The activation of the STAT3 pathway promoted the development of androgen-resistant PRAD (33). There has been evidence that blocking STAT3 activation can reduce inflammatory factors including GM-CSF in animal experiments (34). The chronic inflammatory environment can promote tumor cells or tumor stromal cells to produce inflammatory factors such as GM-CSF; high GM-CSF levels will recruit a large amount of myeloid-derived suppressor cells (MDSCs), while MDSCs in the tumor immune microenvironment has an immunosuppressive function in tumor immunity (35). Therefore, we overexpressed the LTF gene in the PRAD PC-3 cell line and identified the expression of LTF, STAT3, and GM-CSF in PRAD by Western blot analysis, RT-qPCR, and enzyme-linked immunosorbent assay. The experimental results verify our above analysis; that is, overexpression of LTF in PRAD cells can affect the expression of STAT3 through JAK/STAT pathway to reduce tumor-derived GM-CSF secretion and then interfere with the local immunosuppressive function of MDSCs. Thus, LTF has the function of regulating tumor immune microenvironment and inhibiting tumor cell proliferation and migration.

## Conclusion

In summary, we confirmed the changes of immune cell infiltration in the PRAD immune microenvironment, which plays a very important role in the progress of PRAD and provides a theoretical basis for the study of the immune microenvironment mechanism. Also, we identified that LTF is a crucial core gene in the immune microenvironment of PRAD. Its low expression is closely related to poor prognosis. Low expression in tumors will promote primary immunodeficiency and cancer development and is closely related to JAK/STAT signaling pathway. More importantly, we verified through cell experiments that LTF overexpression mediates JAK/STAT pathway to inhibit STAT3 expression and reduce tumor-derived GM-CSF secretion, regulate tumor immune microenvironment, and inhibit tumor cell proliferation and migration.

## Data Availability Statement

The datasets presented in this study can be found in online repositories. The names of the repository/repositories and accession number(s) can be found below: The Cancer Genome Atlas (TCGA; https://portal.gdc.cancer.gov/cart) and Gene Expression Omnibus (GEO) microarray datasets (GSE3325, GSE55945).

## Ethics Statement

The data used in our study were obtained from public databases TCGA and GEO; therefore, ethical approval was not required.

## Author Contributions

QZ and YX contributed to the study design. QZ and YX contributed to data collection and cell experiments. QZ and YC performed statistical analysis and interpretation. QZ drafted the article. YC has verified the underlying data. All authors contributed to the critical revision of the final article and approved the submitted version.

## Funding

This study was supported by the Youth Talents of the Hubei Provincial Health Council (Grant No. WJ2021Q014, WJ2017Q041) and the Department of Health Science, Yangtze University (Grant No. 202003).

## Conflict of Interest

The authors declare that the research was conducted in the absence of any commercial or financial relationships that could be construed as a potential conflict of interest.

## Publisher’s Note

All claims expressed in this article are solely those of the authors and do not necessarily represent those of their affiliated organizations, or those of the publisher, the editors and the reviewers. Any product that may be evaluated in this article, or claim that may be made by its manufacturer, is not guaranteed or endorsed by the publisher.

## Supplementary Material

Supplementary Figure 1 The overall experimental design is shown in , and the experimental design of each part is shown in detail in **Supplementary Figure 2**.

Supplementary Figure 2
**(A)** Bioinformatics method was used to investigate the relationship between the progression of prostate cancer and the immune microenvironment in 591 prostate cancer samples from the TCGA and GEO cohort. **(B)** Identification of genes related to the immune microenvironment in advanced prostate cancer. **(C)** The LTF gene was explored and verified by data samples of 33 common tumor types. **(D)** After overexpression of the LTF gene in PC-3 prostate cancer cells, the expression levels of LTF, JAK, STAT3, and GM-CSF were verified by Western blot.

## Abbreviations

PRAD, prostate cancer; TCGA, The Cancer Genome Atlas; GEO, Gene Expression Omnibus; TMB, tumor mutational burden; MSI, microsatellite instability; GSEA, gene set enrichment analysis; HLA, human leukocyte antigen; PD-L1, programmed death-ligand 1; PD-1, programmed cell death 1; CSF3, colony-stimulating factor 3; LTF, lactoferrin; GM-CSF, granulocyte-macrophage colony–stimulating factor; CRPC, castration-resistant prostate cancer; PAP, prostatic acid phosphatase; TIIC, tumor-infiltrating immune cell; PFI, progression-free interval; KMT2; lysine methyltransferase; STAT3, signal transducer and activator of transcription 3; GEPs, gene expression profiles.
