# Supplementary figures and images for: LTF Regulates the Immune Microenvironment of Prostate Cancer Through JAK/STAT3 Pathway
Source: Front Oncol. 2021 Nov 10;11:692117. doi: 10.3389/fonc.2021.692117 (PMC8635998; doi:10.3389/fonc.2021.692117)

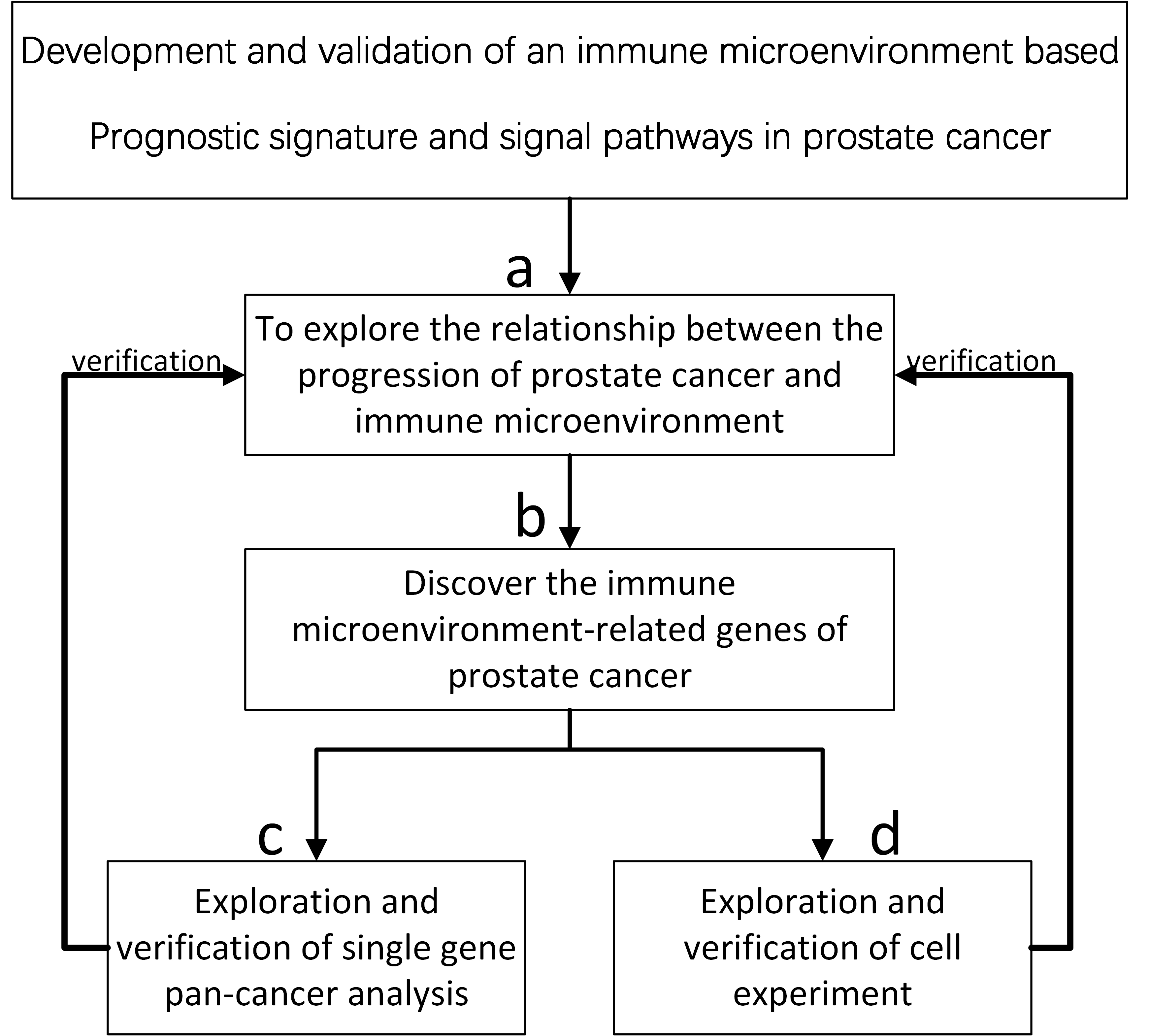

Supplement: Supplementary Figure 1 — The overall experimental design is shown in , and the experimental design of each part is shown in detail in Supplementary Figure 2 . [file Image_1.jpeg]

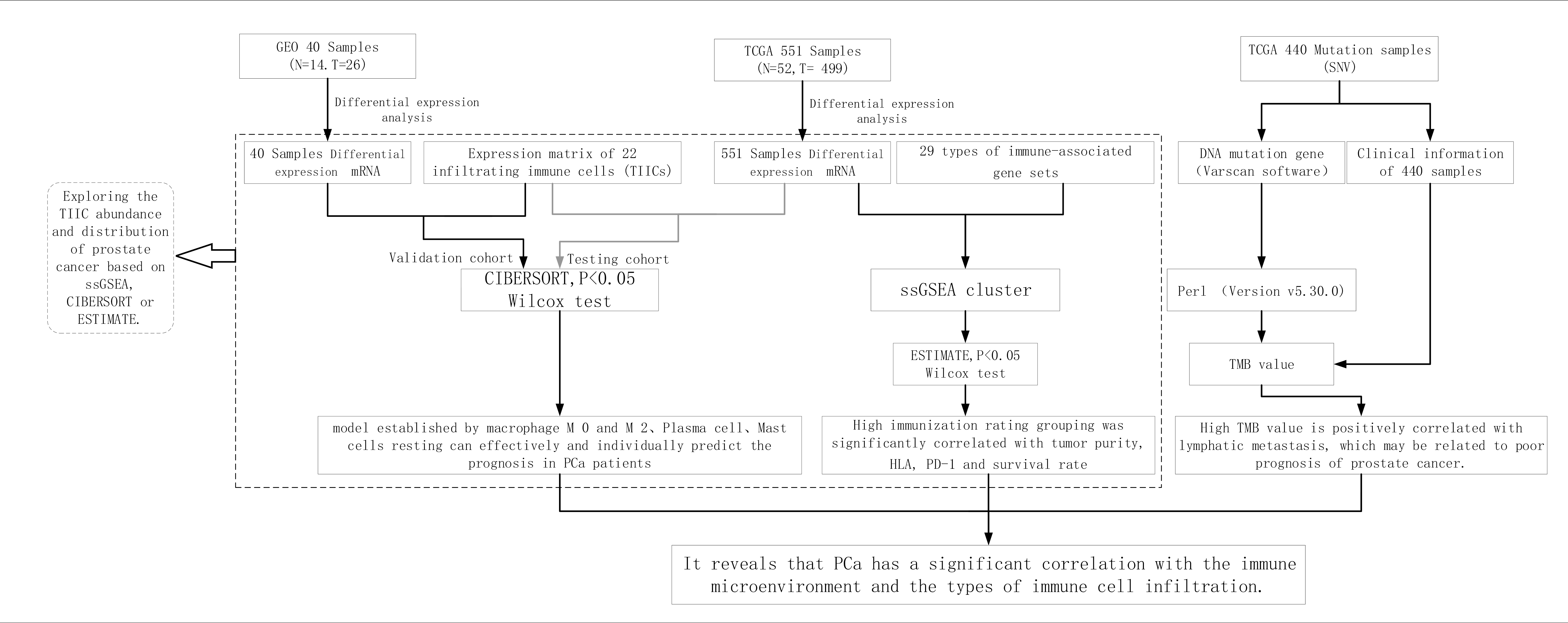

Supplement: Supplementary Figure 2 — (A) Bioinformatics method was used to investigate the relationship between the progression of prostate cancer and the immune microenvironment in 591 prostate cancer samples from the TCGA and GEO cohort. (B) Identification of genes related to the immune microenvironment in advanced prostate cancer. (C) The LTF gene was explored and verified by data samples of 33 common tumor types. (D) After overexpression of the LTF gene in PC-3 prostate cancer cells, the expression levels of LTF, JAK, STAT3, and GM-CSF were verified by Western blot. [file Image_2.jpeg]
